# Development of a clinical diagnostic tool to differentiate multiple myeloma from bone metastasis in patients with destructive bone lesions (MM-BM DDx)

**DOI:** 10.1186/s12875-020-01283-x

**Published:** 2020-10-22

**Authors:** Phichayut Phinyo, Titinat Maihom, Areerak Phanphaisarn, Pakorn Kerdsinchai, Ekarat Rattarittamrong, Jayanton Patumanond, Dumnoensun Pruksakorn

**Affiliations:** 1 Department of Family Medicine, Faculty of Medicine, Chiang Mai University, Chiang Mai, Thailand; 2 Center for Clinical Epidemiology and Clinical Statistics, Faculty of Medicine, Chiang Mai University, Chiang Mai, Thailand; 3 Musculoskeletal Science and Translational Research (MSTR), Chiang Mai University, Chiang Mai, Thailand; 4 Department of Internal Medicine, Faculty of Medicine, Division of hematology, Chiang Mai University, Chiang Mai, Thailand; 5 Biomedical Engineering Institute, Chiang Mai University, Chiang Mai, Thailand; 6 Omics Center for Health Sciences (OCHS), Faculty of Medicine, Chiang Mai University, Chiang Mai, Thailand; 7 Department of Orthopedics, Faculty of Medicine, Orthopedic Laboratory and Research Network (OLARN), Chiang Mai University, Chiang Mai, Thailand

**Keywords:** Bone metastasis, Diagnosis, Multiple myeloma, Osteolytic lesion, Prediction

## Abstract

**Background:**

Most patients with destructive bone lesions undergo a comprehensive diagnostic procedure to ensure that proper treatment decisions are pursued. For patients with multiple myeloma, this can lead to delays in diagnosis and treatment initiation. This study was conducted to develop a diagnostic rule that could serve as a tool for early identification of multiple myeloma and promote timely referral of patients to haematologists.

**Methods:**

The clinical prediction rule was developed using a retrospective case-series of patients with multiple myeloma (MM) and those with bone metastasis (BM) at Chiang Mai University Hospital from 2012 to 2015. Multivariable fractional polynomial logistic regression was used to derive a diagnostic model to differentiate between MM and BM patients (MM-BM DDx).

**Results:**

A total of 586 patients (136 MM patients and 450 BM patients) were included. Serum creatinine, serum globulin, and serum alkaline phosphatase were identified as significant indicators for the differentiation of MM and BM patients. The MM-BM DDx model showed excellent discriminative ability [AuROC of 0.90 (95%CI 0.86 to 0.93)] and good calibration.

**Conclusions:**

This MM-BM DDx model could potentially allow for early myeloma diagnosis and improvement of overall prognosis. A prospective validation study is needed to confirm the accuracy of the MM-BM DDx model prior to its application in clinical practice.

## Background

Clinical approach to the diagnosis of adult patients presenting with a destructive bone lesion is challenging. The differential diagnosis primarily falls between metastatic bone disease and primary bone tumour [1]. Accurate discrimination between those two conditions is of utmost importance as their respective therapeutic goals are different. Usually, these patients undergo numerous non-invasive investigations and consultations aimed at narrowing down the diagnosis without employing invasive procedures. However, in cases where the diagnosis remains uncertain, a tissue biopsy is performed for pathological diagnosis to guide definitive treatment modalities, whether curative or palliative [2]. Surgical stabilization in misclassified primary bone tumours could potentially affect limb salvation and patient survival [1, 3]. For this reason, all patients who present with destructive bone lesions, regardless of the previous history of cancer, must undergo a thorough and often lengthy preoperative evaluation to prevent improper therapeutic decisions [4].

Differentiation of bone metastasis from a primary bone tumor is not the only obstacle clinicians face. Although solid tumours account for a majority of patients with bone metastasis (BM), the differential diagnosis of multiple myeloma (MM) should not be ruled out as there are major distinctions in the overall prognosis and the efficacy of treatment modalities [5]. For example, in the case of BM patients with metastatic spinal cord compression, palliative surgery is a mainstay of treatment in metastatic bone disease with solid origins, whereas in patients with MM, surgical management is not regarded as the primary treatment and should be avoided as these patients respond well to prompt local radiation therapy [2, 6]. However, due to its rarity and nonspecific clinical symptoms, MM is one of the hardest cancers to diagnose [7]. Over half of myeloma patients are subjected to more than three consultations prior to referral to a haematologist [8]. Delayed myeloma diagnosis is reported to be associated with complications and worsening of disease-free survival [9].

Primary care physicians and general orthopedic surgeons who often encounter patients with abnormal bone radiographs play a crucial role in the early identification of MM. Several methods have been proposed to help physicians in the diagnosis of this ambiguous disease at first medical contact, e.g., blood test combinations, electronic trigger-based interventions, and clinical prediction rules [10]. Prompt referral of patients with high pretest probability could increase detection of MM at an early stage, which would subsequently improve the overall outcome of the patients [11]. To date, no clinical prediction rule for the diagnosis of patients with MM has been reported. This study intended to develop and internally validate a simple and practical clinical prediction rule for differential diagnosis of MM from bone metastasis in adult patients presenting with destructive bone lesions which could serve as a supporting tool and as a trigger to clinicians for early referral to specialists.

## Methods

### Study design

Research and development of a diagnostic prediction system including internal validation of a clinical prediction rule was conducted. All study data were retrospectively obtained from Chiang Mai University Hospital electronic medical records from 2012 to 2015. The study was approved by the Institutional Review Board and the Ethics Committee of the Faculty of Medicine, Chiang Mai University.

### Study patients

Case series of patients with MM and patients with bone metastasis diagnosed and treated at Chiang Mai University Hospital within the study period were used for the derivation of diagnostic models. The intended study domain was patients who were suspected by the attending physicians of having either MM or bone metastasis. This group was primarily middle-aged patients presented with bone or back pain and who had abnormal skeletal plain radiographs. However, without problem-oriented medical records, identifying a true ‘intended to be diagnosed’ patient cohort is troublesome in a setting where patients records are tracing based on final diagnosis [12]. For that reason, we applied the method of case-case design and analysis, contrasting clinical characteristics, laboratory values, and bone radiographic patterns of MM case series to bone metastasis case series [13]. We excluded patients aged less than 45 years from the analysis. This exclusion was based on the age distribution of patients with bone metastasis in Thailand, which ranged between 46 and 71 years [14]. The median age of Thai patients with MM was previously reported at 59 years [15].

### Study variables and candidate predictors

Demographic data (age and gender), type of primary cancer, International Staging System (ISS) staging of MM [16], types of paraproteinemia, types of the abnormal bone lesion from plain radiographs, and clinical laboratory values were collected at baseline prior to treatment initiation. Included laboratory variables were hematologic parameters (hemoglobin and hematocrit), renal function test (blood urea nitrogen, serum creatinine (SCr), and serum calcium), liver function test (serum total protein, serum globulin, serum albumin, serum alkaline phosphatase (ALP)), and serum lactate dehydrogenase (LDH). Among all laboratory parameters, we preselected five candidate predictors as follows: hemoglobin, serum creatinine, serum calcium, serum globulin, and serum alkaline phosphatase. The selection of candidate predictors was based on clinical knowledge (i.e., changes in serum globulin level which could reflect abnormal secretion of immunoglobulin from the plasma cell), the classic CRAB (hyperCalcaemia, Renal failure, Anaemia, and Bone lesions) features of MM [17], and other clinical laboratory parameters that are widely used in the detection of bone metastasis (e.g., serum ALP). All plain skeletal radiographs were categorized as either osteolytic lesion, osteoblastic lesion, or mixed lytic-blastic lesion. All the films were reviewed by orthopedic residents and verified by an experienced orthopedic oncologist.

### Clinical endpoints

The diagnosis of both MM and bone metastasis was retrieved from electronic medical records based on ICD-10 (ICD10-C90 MM and ICD10-C795 secondary malignant neoplasm of bone and bone marrow) and subsequently verified by the Chiang Mai Cancer Registry. Myeloma diagnosis was generally based on the standard diagnostic criteria of the International Myeloma Working Group (IMWG), which requires the presence of clonal bone marrow plasma cells ≥10% or biopsy-proven plasmacytoma and at least one myeloma defining event (hypercalcemia, renal insufficiency, anemia, or bone lesions from imaging) [17].

### Statistical methods

#### Fundamental statistical analysis

All statistical analyses were performed using Stata 16 (StataCorp, Lakeway, Texas, USA). Frequency and percentages were used to describe categorical variables. For numerical data, visualization of data distribution was done using histograms. Mean and standard deviation or median and interquartile range were used for the description of continuous variables according to their distributions. Fisher’s exact probability test was used for comparison of categorical variables. t-test and Mann-Whitney test were used for comparison of continuous variables as appropriate. Statistical test results were considered significant if the *p*-values were less than 0.05. Variables with more than 50% missing data were excluded from the analysis.

### Model development

#### Management of missing data

To improve model accuracy, predictive ability, and statistical power, we used multiple imputation with chained equation or MICE for imputation of missing laboratory data values. MM diagnosis and patient demographic data (age and gender) were used as independent variables in predictive mean matching (PMM) methods with *K*-nearest neighbor where *k* = 10 [18]. A total of 10 imputed datasets was derived during these procedures. The differences between incomplete datasets and imputed datasets were evaluated.

#### Handling of continuous predictors

The Transparent Reporting of a multivariable prediction model for Individual Prognosis Or Diagnosis (TRIPOD) statement recommends researchers developing clinical prediction rules avoid categorizing continuous predictors to preserve the completeness of data and the power of the statistics [19]. For this reason, all included clinical laboratory predictors were maintained as continuous. Any predictors with a skewed distribution or which were not normally distributed were converted into a natural logarithmic scale. We explored for possible nonlinear predictor-outcome relationships for each of the laboratory variables using Locally Weighted Scatterplot Smoothing (LOWESS) and fractional polynomials plots. The latter usually provides an optimal model fit through a rich class of simple functions [19]. The best transformation of each predictor was used in the final regression modeling.

#### Multivariable fractional polynomial modeling

The multivariable fractional polynomials or MFP algorithm was applied to fit multiple continuous predictors into a binary logistic regression. The MFP algorithm consists of two steps [20, 21]. The first step is the backward elimination of non-significant predictors from the model. All initially modelled candidate predictors (hemoglobin, log serum creatinine, log serum globulin, serum calcium, and log serum ALP) were tested for their contribution to the model by likelihood-ratio tests or Wald statistics. In this study, a critical alpha level for excluding a predictor from the model was set at 0.2 to reduce the risk of model overfitting. The second step is the iterative approach to identify the best fitting continuous scale for each predictor via a closed test algorithm [22]. The algorithm starts with comparing the best fitting second-degree fractional polynomial (FP2) of each predictor to the null model (without FP2). If the model with FP2 was not significantly superior to the null model, the predictor was excluded from the model. Then, the algorithm compares the FP2 model with the linear model and the FP1 model. If the FP2 model was not superior to the linear model, the linear model was chosen. The FP2 model was selected as the best fitting form only when the FP2 model was superior to the FP1 model in the closed test algorithm. The second cycle of iterations was initiated by fitting a model containing significant covariates with appropriate continuous scales identified from the first cycle. The procedure ceased when two consecutive cycles contained the same set of predictors with the same transformation terms [22].

Multiple imputation with chained equation (MICE) and multivariable fractional polynomial modeling (MFP) was executed via the *mfpmi* function in Stata [23]. The selection of an optimal FP model was based on a version of likelihood-ratio tests modified for multiply imputed data [24]. The model-estimated logit regression coefficients were combined over 10 imputed data sets using Rubin’s rule [25]. As the model was intended to be used by general practitioners or physicians in other specializations who might not feel confident in interpreting abnormal bone lesions in plain skeletal radiographs, only clinical laboratory parameters were included during logistic regression modeling. We also realized that the interpretation of plain skeletal radiographs was, in some circumstances, indefinite and highly varied even among experienced orthopedists and radiologists. Therefore, we did not include types of pathologic appearance from plain film in the model to avoid potential misclassification bias.

#### Study size considerations

We preselected a total of five candidate predictors (hemoglobin level, log serum creatinine, log serum globulin, serum calcium, and log serum ALP) for the diagnostic model. According to previous studies and standard recommendations, it is suggested that a minimum of 10 to 15 clinical endpoint events are needed for each predictor variable included in the logistic regression model [19]. The study requires at least 50 to 75 MM cases are required to minimize the chance of model overfitting.

#### Model performance and internal validation

We measured the diagnostic model performance in terms of discrimination and calibration. The model discriminative ability was evaluated using the area under the receiver operating characteristic curve (AuROC). The model calibration, i.e. the agreement of the model prediction and observed event occurrence, was visualized via a modified calibration plot. As the study base was not a cohort, the probabilities predicted by the model do not reflect the true proportion or risk. For that reason, we exponentiated the linear predictors to derive the model-predicted odds. The model-predicted odds of being diagnosed with MM were then divided into deciles. Next, we graphed the odds curve by plotting the decile mid-points of the model predicted odds on the x-axis and the observed proportions diagnosed with MM within each decile on the y-axis. We also performed statistical tests for calibration using Hosmer-Lemeshow goodness-of-fit. Internal validation was done with a bootstrap re-sampling procedure with 100 replicates. The model optimism and shrinkage factor were estimated and reported.

#### Model presentation and clinical implications

For practicality, the diagnostic model has been developed into a web application. After the input of clinical laboratory parameters, the application shows the predicted odds of a specific patient being MM. To help guide clinicians in decision making, we split the model-predicted odds into deciles. Sensitivity, specificity, and positive likelihood ratios are calculated for each decile of odds. The application finally recommends appropriate further clinical management for each individual patient. Patients with a higher value of likelihood ratio (LHR > 5) should be referred to hematologist for definitive diagnosis of MM. Referral of patients with borderline likelihood ratio values (LHR closes to 1) should be considered on a case-by-case basis based on other relevant clinical parameters, e.g., age, past medical history, and destructive bone pattern. Patients with a high pretest probability of MM should be referred regardless of the model predictions. Referral of patients who are less likely to have MM (LHR below 1) might be withheld; however, regular follow-up visits should be scheduled until a final diagnosis is confirmed.

## Results

**Fig. 1:**
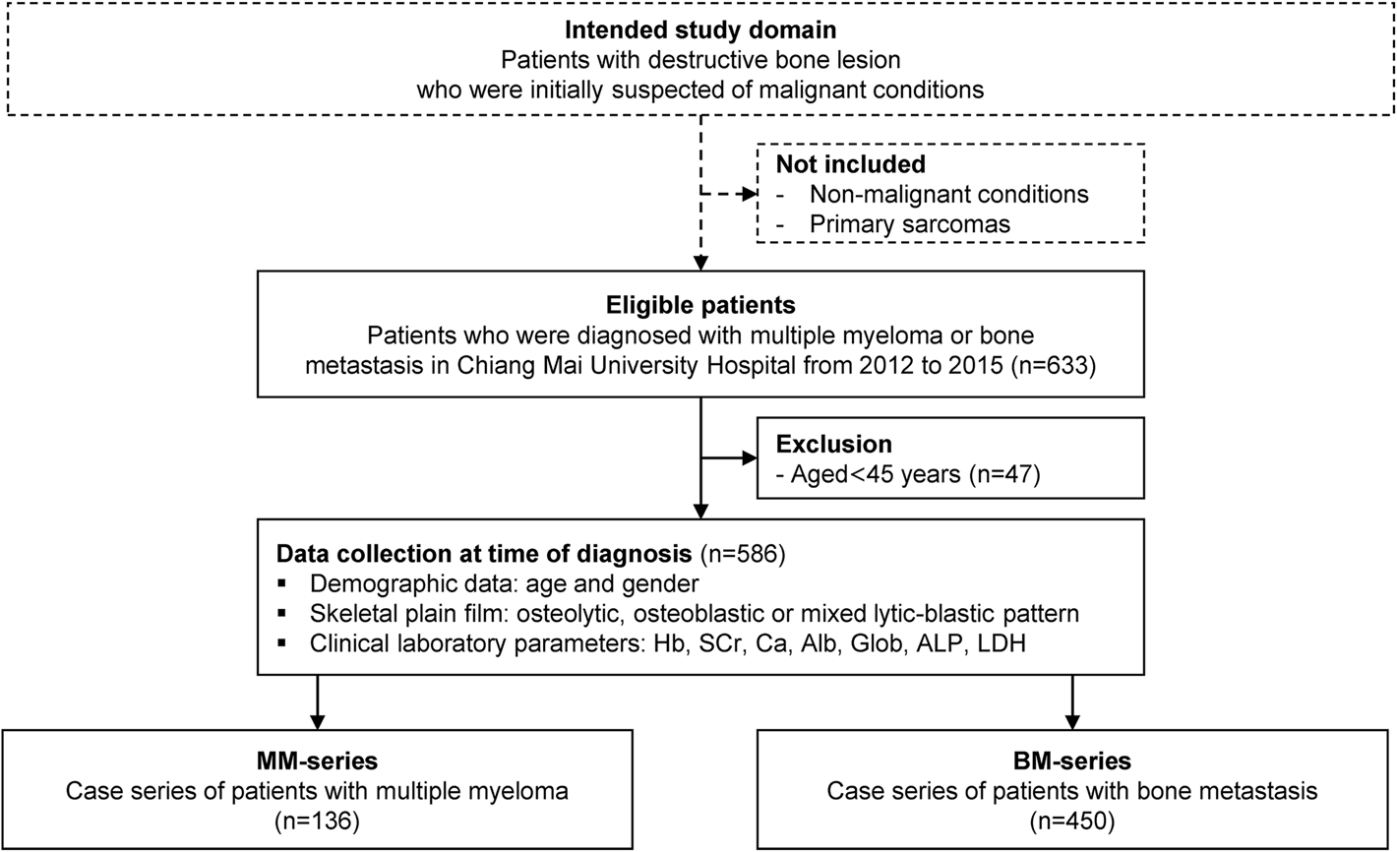
Study flow diagram of MM and BM case-series

From 2012 to 2015, records of 633 patients with MM or bone metastasis diagnosed and treated at Chiang Mai University Hospital were eligible for inclusion. Of that number, 47 patients aged below 45 years were exclude from the analysis. A total of 586 patients, comprising 136 patients with MM and 450 patients with bone metastasis, were used in the derivation of the diagnostic model (Fig. 1). Table 1 presents a comparison of baseline clinical characteristics, abnormal bone radiographic patterns, and clinical laboratory values. The frequency and proportion of missing values for each covariate are also summarized in Table 1. In terms of demographic character, age, and gender did not significantly differ between patients with MM and patients with bone metastasis. Patients with MM and bone metastasis showed statistically significant differences in all clinical laboratory values and in abnormal bone radiographic patterns. Lactate dehydrogenase was omitted from the analysis due to a large proportion of missing values. Due to missing data on serum β2 microglobulin, ISS staging can only be done in 67 (49.3%) of patients with MM. Most patients with MM were found to be diagnosed in the later stages, ISS stages II (22.4%) and III (74.6%) (Additional file 1: Appendix 1). In our study, immunoelectrophoresis results were only available for 115 (84.6%) of patients with MM. IgG was the most common type of serum monoclonal protein (63/115, 54.8%), followed by light chain-only (26/115, 22.6%), IgA (23/115, 20.0%), and alpha heavy chain (1/115, 0.9%). Lung cancer accounted for the highest proportion of patients with bone metastasis (41.8%), followed by liver (13.3%), prostate (9.1%), and breast cancer (7.1%). Supplementary Table, which showed the detail on types and percentages of primary cancer in patients with bone metastasis, was provided (Additional file 1: Appendix 2).

**Table 1:** Baseline clinical characteristics of patients diagnosed with bone metastases or multiple myeloma (*n* = 586)

Clinical character	Missing data		Multiple myeloma (*n* = 136)		Bone metastases (*n* = 450)		*P*-value
	n	(%)	Mean	±SD	Mean	±SD	
**Demographic characteristics**							
Age at presentation (years)	0	(0)	62.4	±9.2	63.2	±9.9	0.417
Gender, (n, %)							
Male	0	(0)	75	(55.2)	253	(56.2)	0.844
Female			61	(44.8)	197	(43.8)	
**Abnormal bone radiographic pattern from plain film**							
No plain film (n, %)	0	(0)	47	(34.6)	204	(45.3)	< 0.001
Osteolytic lesion (n, %)			89	(65.4)	146	(32.4)	
Osteoblastic lesion (n, %)			0	(0)	40	(8.9)	
Mixed lytic-blastic lesion (n, %)			0	(0)	60	(13.3)	
**Clinical laboratory values**							
**Hematologic parameters**							
Hemoglobin (g/dL)	39	(6.7)	9.37	±2.36	11.17	±2.16	< 0.001
Hematocrit (mg %)	44	(7.5)	28.86	±7.48	34.48	±6.38	< 0.001
**Renal function test**							
Blood urea nitrogen (mg/dL, median (IQR))	52	(8.9)	19	(12, 30)	14	(11, 21)	< 0.001
Serum creatinine (mg/dL, median (IQR))	44	(7.5)	1.3	(0.8, 2.3)	0.9	(0.7, 1.2)	< 0.001
Serum calcium (mg/dL)	146	(24.9)	9.81	±1.64	9.21	±1.28	< 0.001
**Liver function test and enzymes**							
Total protein (g/dL)	66	(11.3)	8.82	±2.60	6.96	±0.97	< 0.001
Serum albumin (g/dL)	57	(9.7)	3.10	±0.88	3.42	±0.68	< 0.001
Serum globulin (g/dL)	63	(10.8)	5.66	±3.05	3.56	±1.01	< 0.001
Albumin/Globulin ratio	63	(10.8)	0.80	±0.61	1.04	±0.35	< 0.001
Alkaline phosphatase (U/L, median (IQR))	72	(12.3)	86	(63, 120)	138	(96, 262)	< 0.001
Lactate dehydrogenase (U/L, median (IQR))	511	(86.9)	201	(142, 245)	239	(178, 347)	0.060

Five preselected clinical laboratory result values were included in the multivariable logistic regression model: hemoglobin, log serum creatinine, log serum globulin, serum calcium, and log serum ALP. The fractional polynomials procedure was used for the identification and transformation of nonlinear predictor-outcome relationships. Of the five predictors, only log serum globulin was found to be the best fit with the second-degree fractional polynomial (FP2) and was transformed into FP2 terms for incorporation into the model. The rest of the predictors were included as linear terms with mean subtractions. Hemoglobin and serum calcium were omitted from the model due to the insignificant association. The covariate transformations, logit regression coefficients with 95% confidence intervals, and their *p*-values are presented in Table 2. Supplementary Table in the appendix shows the closed test algorithm of the multivariable fractional polynomial logistic regression model (Additional file 1: Appendix 3).

**Table 2:** Multivariable fractional polynomial logistic regression model for diagnostic prediction of multiple myeloma. (imputed dataset with a total *n* = 586)

Predictor	Covariate transformation			ß	95% CI	*P*-value
	Terms	df	Formula			
Intercept				−2.28	−2.63, −1.93	< 0.001
Hemoglobin	Out	0	–	–	–	–
Log serum creatinine	Linear	1	Log creatinine-0.0237	1.28	0.80, 1.75	< 0.001
Log serum globulin	Linear	4	Log globulin^**-0.5**^-0.8714	− 92.64	− 114.80, −70.49	< 0.001
	FP2		Log globulin^-0.5^*^Log (Log globulin)^-0.2400	−48.14	−60.13, −36.15	< 0.001
Log alkaline phosphatase	Linear	1	Log ALP-4.9318	−0.97	−1.38, −0.56	< 0.001
Serum calcium	Out	0	–	–	–	–

*Abbreviations*: *df* Degrees of freedom, *CI* Confidence interval, *Log* Natural logarithm function, *FP2* Second-degree fractional polynomial, *ALP* Alkaline phosphates

**Fig. 2:**
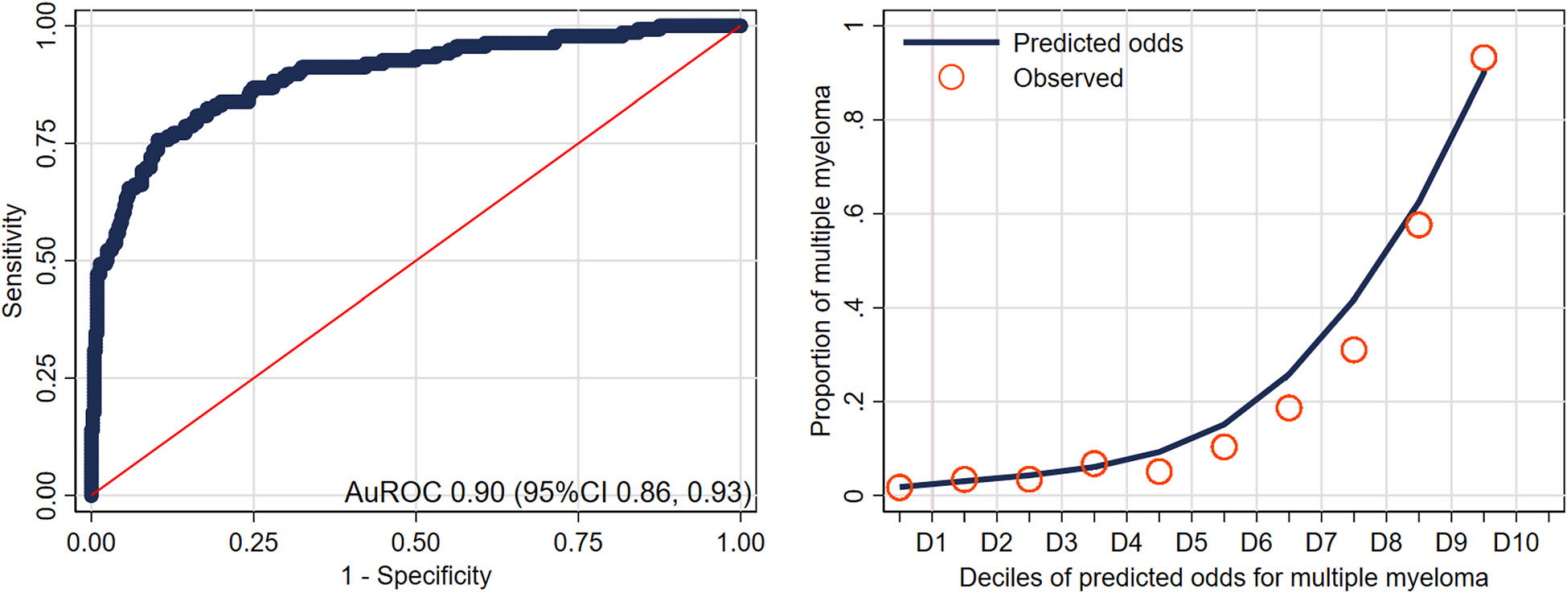
**a** Receiver operating characteristics curve (ROC) of the diagnostic prediction model for multiple myeloma based on clinical laboratory values. AuROC 0.90 (95%CI 0.86–0.93) **b** Model calibration plot of the agreement between model predicted odds and observed proportion of patients with multiple myeloma for each decile of predicted odds

The derived diagnostic model (MM-BM DDx) showed excellent discriminative performance with an AuROC of 0.90 (95%CI 0.86 to 0.93) (Fig. 2a). The model calibration was good, as evident from the calibration plot comparing model-predicted odds and observed proportion of MM within each specific decile (Fig. 2b). The Hosmer-Lemeshow goodness-of-fit statistic was insignificant (*P* = 0.960). Internal validation via bootstrap sampling showed a consistent AuROC of 0.90 (95%CI 0.89 to 0.90) with minimal model optimism at 0.003 (range − 0.043, 0.041). The shrinkage factor was estimated to be 1.07 (95%CI 0.96 to 1.19) (Additional file 1: Appendix 4).

The predicted odds of MM from the model were split into deciles of odds (D1 to D10). The frequency of patients with MM increased as the number of deciles increased. Table 3 presents the median and range of model-predicted odds of MM for each odds decile and the decile-specific diagnostic indices (sensitivity, specificity, and positive likelihood ratio) for identifying appropriate cutoffs.

**Table 3:** Diagnostic accuracy of the MM-BM DDx model by deciles. This table showed sensitivity, specificity, and positive likelihood ratio at each decile of model-predicted odds of multiple myeloma from clinical laboratory values

Decile	n	MM	BM	Median predicted odds	Range of predicted odds (min, max)	Sensitivity (95% CI)	Specificity (95% CI)	LHR+ (95% CI)
D1	58	1	57	0.016	0.003, 0.027	100.0 (97.3, 100.0)	0 (0, 0.02)	0.06 (0.00, 0.34)
D2	59	2	57	0.037	0.027, 0.046	99.3 (96.0, 100.0)	12.7 (9.7, 16.1)	0.11 (0.01, 0.45)
D3	58	2	56	0.057	0.046, 0.067	97.8 (93.7, 99.5)	25.3 (21.4, 29.6)	0.11 (0.01, 0.46)
D4	59	4	55	0.075	0.067, 0.087	96.3 (91.6, 98.8)	37.8 (33.3, 42.4)	0.24 (0.06, 0.67)
D5	59	3	56	0.104	0.088, 0.118	93.4 (87.8, 96.9)	50.0 (45.3, 54.7)	0.18 (0.04, 0.56)
D6	58	6	52	0.129	0.118, 0.150	91.2 (85.1, 95.4)	62.4 (57.8, 66.9)	0.38 (0.13, 0.92)
D7	59	10	49	0.183	0.152, 0.227	86.8 (79.9, 92.0)	74.0 (69.7, 78.0)	0.68 (0.30, 1.40)
D8	58	18	40	0.356	0.229, 0.526	79.4 (71.6, 85.9)	84.9 (81.2, 88.1)	1.49 (0.78, 2.76)
D9	59	35	24	1.257	0.543, 3.222	66.2 (57.6, 74.1)	93.8 (91.1, 95.8)	4.83 (2.68, 8.77)
D10	59	55	4	14.864	3.330, 3111.405	40.4 (32.1, 49.2)	99.1 (97.7, 99.8)	45.50 (16.26, 174.85)
Total	586	136	450					

*Abbreviations*: *MM* Multiple myeloma, *BM* Bone metastasis, *min* Minimum, *max*, Maximum, *CI* Confidence interval, *LHR+* Positive likelihood ratio, *D* Decile

An online web application for calculation of the model predicted odds of MM in patients who present with destructive bone lesions is available at: https://www.calconic.com/calculator-widgets/mm-bm-ddx-by-med-cmu/5e05f974471eb4001e99baf8.

## Discussion

Malignant destructive bone lesion usually presents in one of three forms: isolated osteolytic, isolated osteoblastic, or mixed osteolytic-osteoblastic lesions. In a middle-aged patient, secondary bone metastasis is the most common and can appear in any radiologic image, with the appearance dependent on the type of primary cancer [26]. Conversely, bone lesions found with MM are distinct from those of other malignancies [27]. In MM, the typical appearance is purely osteolytic, resulting from an increase in osteoclastic (OCL) activity and a marked decrease in osteoblastic (OBL) activity [28]. Although osteoblastic and mixed lesions are rarely found in MM, clinicians should always consider the possibility of MM, even in the presence of diffuse bone sclerosis [29].

In practice, both MM and bone metastasis usually present with similar radiographic findings, which are hard to classify accurately, even for experienced radiologists [30]. Some advanced imaging modalities, such as magnetic resonance imaging (MRI), were suggested to be used for differentiation of MM bone lesions from those of metastatic cancer. However, even with MRI, special techniques must be applied for accurate interpretation [30, 31]. That indicates relying solely on radiographic findings for the diagnosis of patients with destructive bone lesions is not adequate. Other relevant clinical parameters should be incorporated into the diagnostic process of this group of patients.

In Thailand, general physicians or general orthopedic surgeons are usually the first to encounter patients with destructive bone lesions on plain radiographs. Generally, clinical judgment based on available patient profiles was used to make referral decisions, either to haematologists or orthopedic oncologists. Regarding MM, most physicians would consider the CRAB criteria to rule in MM diagnosis. However, differentiation of MM from bone metastasis can be difficult, as advanced-stage cancer patients usually have several organ dysfunctions which result in abnormal laboratory values that can mimic all the cardinal features of MM, e.g., anaemia [32], impaired renal function [33], and hypercalcemia [34]. Therefore, it is unlikely that using the CRAB criteria to differentiate patients suspected of bone metastasis from patients with MM would be appropriate. Moreover, the CRAB criteria were not originally developed to be used for this purpose, but to be used to distinguish symptomatic MM from its precursor states, such as monoclonal gammopathy of undetermined significance (MGUS) and smoldering multiple myeloma (SMM) [35].

We have yet to find any other diagnostic models or algorithms that directly and specifically answer our objective. Some approaches might seem relevant but could not be directly applied to our situation [11, 36–38], as most primarily intended to screen patients for early MM diagnosis not to differentiate MM from bone metastasis. The concept to differentiate BM and MM from one another in adult patients presented with destructive bone lesion was often overlooked and left to each physician’s discretion. By utilizing the multivariable regression approach and fractional polynomial procedure, we were able to model all the continuous laboratory values according to their relationships with the log odds of having MM and develop a novel diagnostic approach to differentiate adult patients who presented with destructive bone lesion with only a few simple, routinely available laboratory tests: serum creatinine, serum globulin, and serum ALP. To the best of our knowledge, this is the first clinical prediction rule to address the differential diagnosis of MM from bone metastasis in patients who present with malignant bone lesions.

All of the predictors included in the model can be justified based on the pathophysiological processes associated with the two diseases. In terms of impaired renal function, MM is widely regarded as an important cause of cancer-related end-stage renal disease (ESRD) [39]. On average, 20 to 30% of patients with MM are reported to have renal failure upon diagnosis, which is associated with shorter survival [40]. MM causes severe injury to the kidney via various pathways, most commonly through myeloma cast nephropathy (MCN) caused by the accumulation of free immunoglobin light chains within renal tubules [41]. For other solid tumors, acute kidney injury can result from pre-renal (e.g., intravascular volume depletion, chronic blood loss, sepsis), intrinsic (e.g., tubulointerstitial or glomerular pathology), or post-renal obstruction (e.g., bladder or ureter obstruction) [42]. Again, the spectrum of renal failure varies across different types of cancer. It has been reported that at the time of diagnosis, a significantly larger proportion of patients with MM (48%) have abnormal serum creatinine compared to patients with other cancers (3%) both with and without bone metastasis [33], which is consistent with the proportions identified from our data (49.7% in MM vs. 20.4% other cancers). Thus, in our model, patients with higher levels of initial serum creatinine would be shifted towards a diagnosis of MM.

Serum protein components (e.g., albumin-to-globulin ratio or AGR) have been used as a clinical indicator of various conditions, including MM, immunoproliferative diseases, and other malignant conditions [43]. Low AGR has been reported to be linked to carcinogenesis and elevated markers of chronic inflammation [44]. In MM, the neoplastic proliferation of plasma cells results in an overproduction of monoclonal immunoglobulins, which can increase serum globulin. However, a high level of serum globulin alone does not signify the presence of MM as the abnormalities could be either monoclonal (e.g., monoclonal gammopathy) or polyclonal (e.g., cancer, chronic infections, connective tissue disorders, or liver disease) [45]. A fraction of patients with myeloma, light chain secreting disease, or a low or non-secreting disease [46] might have low or normal levels of serum globulin [15]. This was clearly reflected in our data via fractional polynomial plots (Additional file 1: Appendix 5). From the model, serum globulin was identified as the strongest of the predictors. The serum globulin level in patients with MM was significantly higher than that of patients with bone metastasis. According to our data, no patients with bone metastasis had serum globulin exceeding 12 mg/dL.

Serum ALP is a hydrolase enzyme which is secreted by various organs including the liver, intestine, and placenta. Bone ALP is one of the isoforms of ALP that is specifically present on the surface of osteoblasts. Serum level of bone ALP has been shown to have a positive correlation with osteoblastic activity or bone formation [47]. Therefore, ALP is generally considered as a marker of bone turnover. In patients with bone metastasis, ALP was found to be significantly higher than normal, especially in cases of blastic disease [48]. For this reason, serum ALP has been used widely for monitoring cancer patients for bone metastasis and, in some circumstances, it can also be used to predict the survival outcomes of patients [49]. On the other hand, despite an increase in osteolytic activity, osteoblastic activity is severely suppressed in MM [28]. One study reported that ALP levels were significantly higher in patients with breast and prostate cancer than in patients with MM [50], which is in concordance with our study. Therefore, patients with high serum ALP levels would be more likely to have bone metastasis than MM.

Hemoglobin and serum calcium was not an independent predictor of MM in the diagnostic model despite the significant univariable comparisons. Both MM and bone metastasis can exhibit anaemia and hypercalcemia through various mechanisms. In this study, the degree of anaemia in advanced-stage patients with bone metastasis is less severe compared to patients with MM. According to previous reports, less than one-third of patients with a solid tumor had bone marrow metastasis, while almost all patients with MM required the presence of marrow invasion by the cancer cells to fulfil the standard diagnostic criteria [17, 51]. Nearly half of the patients with bone metastasis in our series had lung and/or breast cancer, the common etiology of malignancy-associated hypercalcemia. Even though MM had the highest rate of hypercalcemia when compared to other malignancies as reported in a US study [34], the magnitude of the association was minimal compared to other predictors in the model. Insignificance association of both hemoglobin and serum calcium supported our prior hypothesis that using CRAB criteria for proper diagnosis might not be appropriate in this context.

The present study did not include some of the proposed clinical predictors for early detection of MM such as plasma viscosity, erythrocyte sedimentation rate (ESR), and C-reactive protein (CRP) [36] as our study was not intended to screen general patients for MM, but rather to improve accuracy in the differentiation of patients with MM from patients with bone metastasis. In addition, in most settings, the equipment needed to measure plasma viscosity is not available, and obtaining the necessary equipment would not be cost-effective. ESR and CRP are also widely known as being unspecific for diagnosis and can be interfered with many factors. The overall diagnostic utility of those three tests has also been found to be similarly low [52]. In addition, the inclusion of other inflammatory markers in the model would have been inappropriate due to the risk of collinearity with serum globulin.

**Fig. 3:**
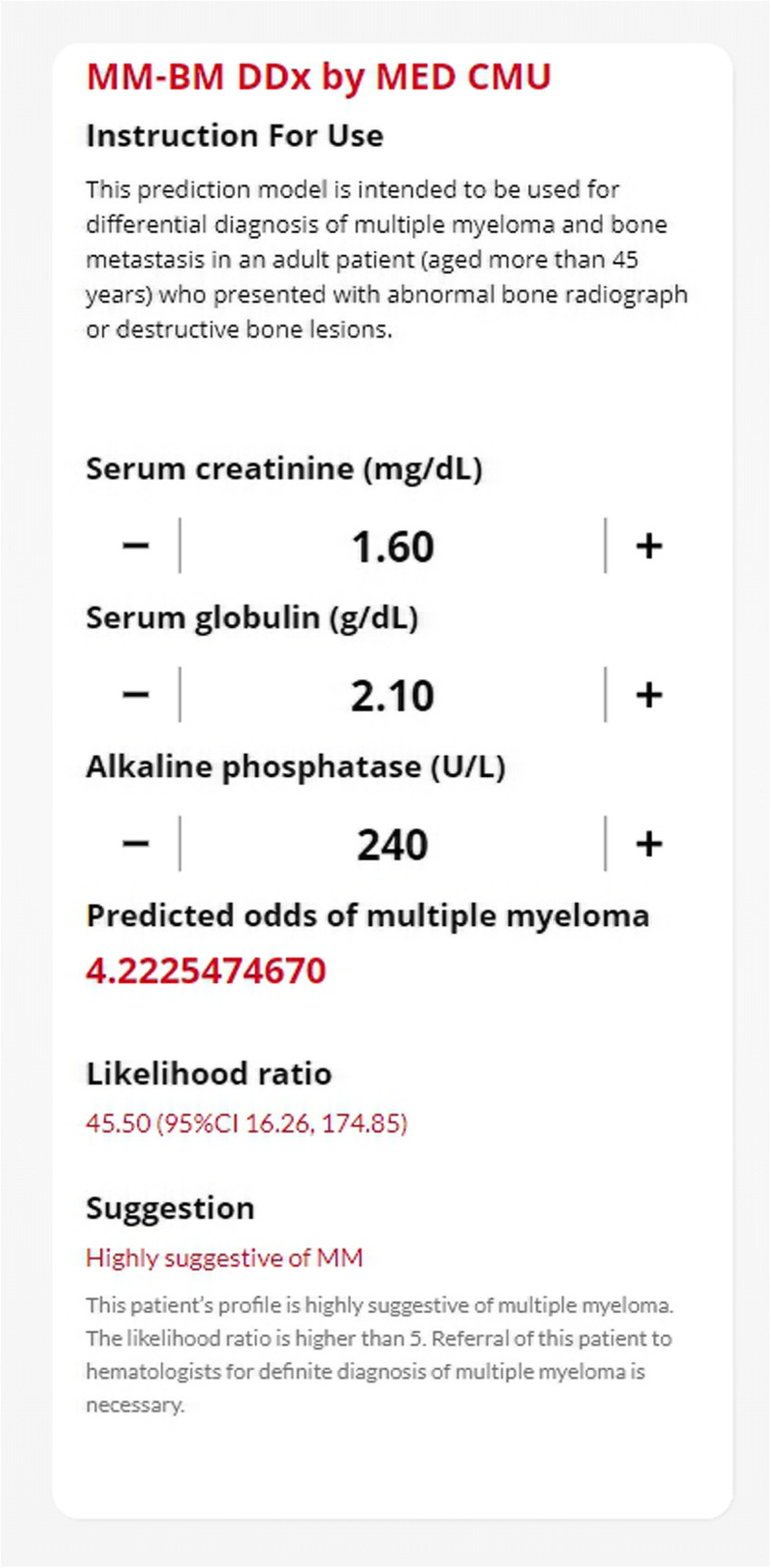
The web application interface of the MM-BM DDx model. Three clinical laboratory parameters can be used for prediction of the presence of multiple myeloma

The clinical application of the proposed diagnostic model is simple and straightforward. The model should be used specifically in patients who present with abnormal destructive bone lesions identified from imaging regardless of whether they have previously been diagnosed with other cancers. To demonstrate the model’s value, we applied our model to the case report of a 65-year-old male patient who had been recently diagnosed with adenocarcinoma of the colon [53]. About 5 months after primary cancer resection, the patient returned with low back pain and weight loss. Physical examination showed tenderness over the thoracic vertebrae. Skeletal radiographs revealed osteoporosis with a compression fracture in the thoracolumbar region and several osteolytic lesions in the skull. Based on the patient’s medical history, it would be reasonable to suspect secondary bone metastasis based on the higher prevalence of the disease as synchronous MM is rare. The initial laboratory workup of the patient was as follows: hemoglobin 10 g/dL, serum creatinine 1.6 mg/dL, serum albumin 4.3 g/dL, serum globulin 2.1 g/dL, and serum ALP 240 IU/L. The model predicted the odds of MM at 4.223 with a positive likelihood ratio of 45.50 (highly suggestive of MM) (Fig. 3). Based on that, this patient should have been referred to a hematologist for further work-up and a definitive diagnosis. This was a case of coexistence of colon carcinoma and non-secreting MM. During some circumstances, timely referral of MM patients to haematologists is necessary for favorable outcomes. In our experience, we had encountered a patient with destructive bone lesions at the thoracolumbar spine who presented with acute spinal cord compression. For decompressive surgery, MRI of the spine for the identification of compressive sites was urgently requested. The patient also underwent primary cancer identification via several other imaging modalities. While the results were expected within a few days, simple laboratory parameters (including serum creatinine, serum globulin, and serum ALP) were available within a few hours after admission. Recognizing the abnormal values of those parameters, we transferred the patient to the haematology department, where MM was subsequently diagnosed. The patient timely received emergency radiotherapy, which effectively improves the patient’s paralysis.

Our study includes both strengths and limitations. Among the strengths, we did not categorize any of the clinical laboratory values before their inclusion in the model to prevent the loss of detailed information and obliteration of the true shape of the predictor-outcome association. Also, the use of the fractional polynomial approach allowed us to capture nonlinear associations during the modeling process, which improves the model accuracy. Lastly, all the predictors were based on laboratory investigations that are inexpensive and readily-available, even in primary care and resource-limited settings. The first limitation is that the model was developed from a retrospective database obtained from a single institution. Second, the study was not based on an entire cohort of patients who presented with abnormal bone radiographs, but rather a sample of case-series of patients with MM and case-series of patients with bone metastasis. Additionally, patients with benign conditions and primary sarcoma were not included in the analysis. Because of those limitations, the model was not able to accurately predict the probability of having MM. For that reason, predicted odds of MM and positive likelihood ratios were reported instead of probabilities. The use of predicted odds and likelihood ratios might cause confusion due to unfamiliarity of the concept among users. To avoid that situation, pre-specified clinical suggestions have been inserted to elaborate the model prediction to guide proper management by the user. Another limitation is that owing to the retrospective nature of the data, information on β2 microglobulin for ISI staging and types of monoclonal protein was available for only a fraction of the patients with MM. However, it was observed that the pattern of staging did not vary for different years. That indicates the missing data were likely random, and the pattern may reflect the underlying distribution of staging in patients with MM. Finally, the application of the model in a population with different case mixes and disease spectrums might negatively impact the model’s overall performance. Although the model’s discriminative ability was shown to be robust based on internal validation, a prospective external validation study, both narrow (nationally) and broad (internationally), is necessary before the model is launched for clinical use.

## Conclusions

A diagnostic model for the differentiation of MM from bone metastasis in patients who present with destructive bone lesions, the MM-BM DDx model, was developed. By including only routinely available clinical laboratory values, the MM-BM DDx model provides accurate predictions for individual patients and facilitates timely referral of patients with a high likelihood of MM to hematologists. The application of the MM-BM DDx model in clinical practice could potentially increase early myeloma diagnosis and, as a consequence, improve the overall survival of the patients.

## Supplementary information

**Additional file 1: Appendix 1.** International Staging System (ISS) staging of patients with multiple myeloma. **Appendix 2.** Types of primary cancer in patients diagnosed with bone metastasis. **Appendix 3.** Closed test algorithm of multivariable fractional polynomial logistic regression model via *mfpmi* package using an imputed dataset (*n* = 586). **Appendix 4.** Internal validation via bootstrap procedures with 100 replicates. **Appendix 5.** Locally-weighted scatter plot smoothing (LOWESS) and fractional polynomial assessment of linear association between clinical laboratory predictors and log odds of multiple myeloma: (a) hemoglobin, (b) log serum creatinine, (c) log serum globulin, (d) serum calcium, and (e) log alkaline phosphatase.

## Data Availability

The datasets used and/or analysed during the current study are available from the corresponding author on reasonable request.

## Abbreviations

MM: Multiple myeloma
BM: Bone metastasis
ISS: International staging system
Hb: Hemoglobin
Hct: Hematocrit
SCr: Serum creatinine
Ca: Serum calcium
ALP: Alkaline phosphatase
LDH: Lactate dehydrogenase
ICD: International classification of disease
IMWG: International myeloma working group
MICE: Multiple imputation with chained equation
PMM: Predictive mean matching
TRIPOD: Transparent reporting of a multivariable prediction model for individual prognosis or diagnosis
LOWESS: Locally weighted scatter plot smoothing
MFP: Multivariable fractional polynomial
FP: Fractional polynomial
AuROC: Area under receiver operating characteristic curve
LHR: Likelihood ratio
IgG: Immunoglobulin G
IgA: Immunoglobulin A
D: Decile
OCL: Osteoclastic
OBL: Osteoblastic
MGUS: Monoclonal gammopathy of undetermined significance
SMM: Smoldering multiple myeloma
ESRD: End-stage renal disease
MCN: Myeloma cast nephropathy
AGR: Albumin-to-globulin ratio
CRP: C-reactive protein
ESR: Erythrocyte sedimentation rate
