# Correction: Development of a clinical diagnostic tool to differentiate multiple myeloma from bone metastasis in patients with destructive bone lesions (MM-BM DDx)

**DOI:** 10.1186/s12875-022-01785-w

**Published:** 2022-07-28

**Authors:** Phichayut Phinyo, Titinat Maihom, Areerak Phanphaisarn, Pakorn Kerdsinchai, Ekarat Rattarittamrong, Jayanton Patumanond, Dumnoensun Pruksakorn

**Affiliations:** 1 Department of Family Medicine, Faculty of Medicine, Chiang Mai University, Chiang Mai, Thailand; 2 Center for Clinical Epidemiology and Clinical Statistics, Faculty of Medicine, Chiang Mai University, Chiang Mai, Thailand; 3 Musculoskeletal Science and Translational Research (MSTR), Chiang Mai University, Chiang Mai, Thailand; 4 Department of Internal Medicine, Faculty of Medicine, Division of hematology, Chiang Mai University, Chiang Mai, Thailand; 5 Biomedical Engineering Institute, Chiang Mai University, Chiang Mai, Thailand; 6 Omics Center for Health Sciences (OCHS), Faculty of Medicine, Chiang Mai University, Chiang Mai, Thailand; 7 Department of Orthopedics, Faculty of Medicine, Orthopedic Laboratory and Research Network (OLARN), Chiang Mai University, Chiang Mai, Thailand

**Correction: BMC Primary Care 21, 215 (2020)**

10.1186/s12875-020-01283-x

Following publication of the original article [1], the authors identified some errors in the published equation on Table 2, the published Fig. 3 (the estimated odds and likelihood ratio were incorrect due to our mistake in specifying the equation within the calculator), and the discussion part (related to the figure) in the manuscript. The correction details are stated as follows:

1. Log (Log globulin) of the 4^th^ entry under Formula column should not be superscripted.

**Table 2:** Multivariable fractional polynomial logistic regression model for diagnostic prediction of multiple myeloma. (imputed dataset with a total *n* = 586)

Predictor	Covariate transformation			ß	95% CI	*P*-value
	Terms	df	Formula			
Intercept				-2.28	-2.63, -1.93	<0.001
Hemoglobin	Out	0	-	-	-	-
Log serum creatinine	Linear	1	Log creatinine-0.0237	1.28	0.80, 1.75	<0.001
Log serum globulin	Linear	4	Log globulin^**-0.5**^-0.8714	-92.64	-114.80, -70.49	<0.001
	FP2		Log globulin^-0.5^*Log (Log globulin)-0.2400	-48.14	-60.13, -36.15	<0.001
Log alkaline phosphatase	Linear	1	Log ALP-4.9318	-0.97	-1.38, -0.56	<0.001
Serum calcium	Out	0	-	-	-	-

2) Our previous equation embedded within the online web calculator was incorrect. After correction, the model estimated the predicted odds of MM at 0.436 with a positive likelihood ratio of 1.429 (suspected MM) (Fig. 3). Figure 3 was corrected accordingly. The suggestion in the following sentence was also corrected to follow the model’s estimates. The 9^th^ and 10^th^ sentence of the 3^rd^ paragraph on page 9 should read:

The model predicted the odds of MM at 0.436 with a positive likelihood ratio of 1.429 (suspected MM) (Fig. 3). Based on that, this patient should have been considered for referral to a hematologist for further work-up and a definitive diagnosis.

**Fig. 3:**
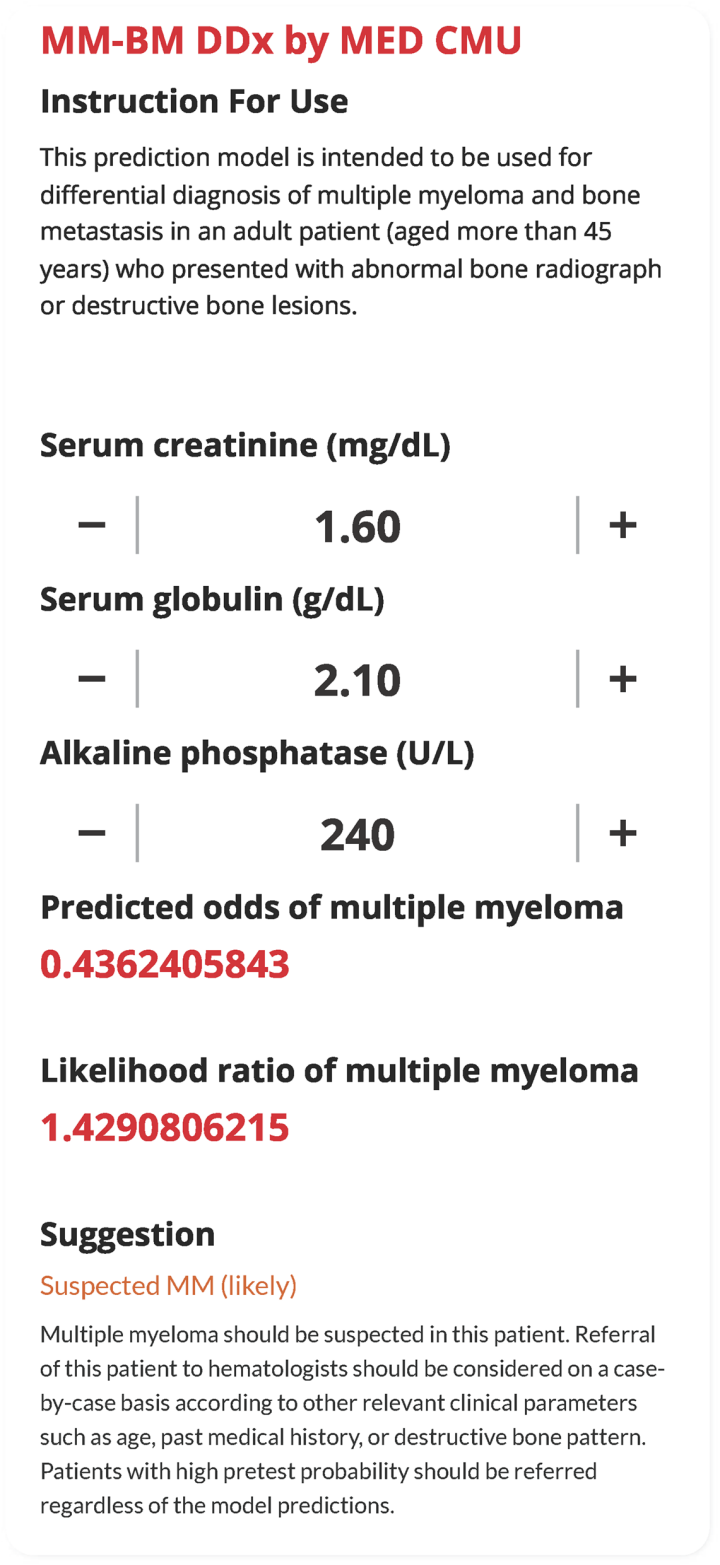
The web application interface of the MM-BM DDx model. Three clinical laboratory parameters can be used for prediction of the presence of multiple myeloma

## References

[1] Phinyo P, Maihom T, Phanphaisarn A (2020). Development of a clinical diagnostic tool to differentiate multiple myeloma from bone metastasis in patients with destructive bone lesions (MM-BM DDx). BMC Fam Pract.

