# Supplementary material for: Development of a clinical diagnostic tool to differentiate multiple myeloma from bone metastasis in patients with destructive bone lesions (MM-BM DDx)
Source: BMC Fam Pract. 2020 Oct 22;21:215. doi: 10.1186/s12875-020-01283-x (PMC7579980; doi:10.1186/s12875-020-01283-x)

**Appendix 1.** International Staging System (ISS) staging of patients with multiple myeloma

| Year | International Staging System (ISS) staging | | | | | | Total |
| --- | --- | --- | --- | --- | --- | --- | --- |
|  | Stage I | | Stage II | | Stage III | |  |
|  | n | (%) | n | (%) | n | (%) |  |
| 2012 | 0 | (0) | 4 | (33.3) | 8 | (66.7) | 12 |
| 2013 | 0 | (0) | 2 | (11.8) | 15 | (88.2) | 17 |
| 2014 | 1 | (5.0) | 7 | (35.0) | 12 | (60.0) | 20 |
| 2015 | 1 | (5.6) | 2 | (11.1) | 15 | (83.3) | 18 |
| Total | 2 | (3.0) | 15 | (22.4) | 50 | (74.6) | 67 |

**Appendix 2.** Types of primary cancer in patients diagnosed with bone metastasis

| Primary cancer | ICD-10 | Total (n=450) | |
| --- | --- | --- | --- |
|  |  | n | (%) |
| Lung | C34 | 188 | (41.8) |
| Liver | C22 | 60 | (13.3) |
| Prostate | C61 | 41 | (9.1) |
| Breast | C50 | 32 | (7.1) |
| Thyroid | C73 | 11 | (2.4) |
| Stomach | C16 | 10 | (2.2) |
| Pancreas | C25 | 6 | (1.3) |
| Urinary bladder | C67 | 6 | (1.3) |
| Lymphoma | C81-C96 | 6 | (1.3) |
| Nasopharynx | C11 | 5 | (1.1) |
| Colon | C18 | 5 | (1.1) |
| Cervix | C53 | 4 | (0.9) |
| Connective tissue | C48 | 2 | (0.4) |
| Kidney | C64 | 2 | (0.4) |
| Rectosigmoid | C19 | 1 | (0.2) |
| Skin | C44 | 1 | (0.2) |
| Brain | C71 | 1 | (0.2) |
| Spinal cord | C72 | 1 | (15.1) |
| Unknown |  | 68 |  |

**Appendix 3.** Closed test algorithm of multivariable fractional polynomial logistic regression model via *mfpmi* package using an imputed dataset (n=586)

|  | **Variable** | **Model** | | **Deviance** | **P-value** | **Power** | **(vs.)** |
| --- | --- | --- | --- | --- | --- | --- | --- |
|  | Log serum globulin | Null | FP2 | 87.414 | <0.001 | . | -0.5 -0.5 |
|  |  | Linear |  | 60.652 | <0.001 | 1 |  |
|  |  | FP1 |  | 41.737 | <0.001 | 3 |  |
|  |  | Final |  |  |  | -0.5 -0.5 |  |
|  | Log serum creatinine | Null | FP2 | 20.336 | <0.001 | . | 3 3 |
|  |  | Linear |  | 0.364 | 0.948 | 1 |  |
|  |  | Final |  |  |  | 1 |  |
|  | Hemoglobin | Null | FP2 | 2.538 | 0.638 | . | 3 3 |
|  |  |  |  |  |  |  |  |
|  |  | Final |  |  |  | . |  |
|  | Log alkaline phosphatase | Null | FP2 | 41.798 | <0.001 | . | 0 0 |
|  |  | Linear |  | 5.602 | 0.133 | 1 |  |
|  |  | Final |  |  |  | 1 |  |
|  | Serum calcium | Null | FP2 | 3.161 | 0.531 | . | 3 3 |
|  |  | Final |  |  |  | . |  |
| **End of cycle 1** | | | | | | | |
|  | **Variable** | **Model** | | **Deviance** | **P-value** | **Power** | **(vs.)** |
|  | Log serum globulin | Null | FP2 | 118.131 | <0.001 | . | -0.5 -0.5 |
|  |  | Linear |  | 73.422 | <0.001 | 1 |  |
|  |  | FP1 |  | 49.061 | <0.001 | 3 |  |
|  |  | Final |  |  |  | -0.5 -0.5 |  |
|  | Log serum creatinine | Null | FP2 | 32.777 | <0.001 | . | 3 3 |
|  |  | Linear |  | 0.592 | 0.898 | 1 |  |
|  |  | Final |  |  |  | 1 |  |
|  | Hemoglobin | Null | FP2 | 2.240 | 0.692 | . | 3 3 |
|  |  |  |  |  |  |  |  |
|  |  | Final |  |  |  | . |  |
|  | Log alkaline phosphatase | Null | FP2 | 28.170 | <0.001 | . | 1 1 |
|  |  | Linear |  | 4.809 | 0.186 | 1 |  |
|  |  | Final |  |  |  | 1 |  |
|  | Serum calcium | Null | FP2 | 3.161 | 0.531 | . | 3 3 |
|  |  | Final |  |  |  | . |  |
| **End of cycle 2** | | | | | | | |
| *Fractional polynomial fitting algorithm converged after 2 cycles | | | | | | | |

Abbreviations: Log, natural logarithm function; FP1, first-degree fractional polynomial; FP2, second-degree fractional polynomial, (vs)., comparison of polynomial terms.

**Appendix 4.** Internal validation via bootstrap procedures with 100 replicates

|  | **Observed**  **Coefficients** | **Bootstrap**  **Standard error** | **z-score** | **P-value** | **Normal-based** | |
| --- | --- | --- | --- | --- | --- | --- |
|  |  |  |  |  | **95% Confidence Interval** | |
| Apparent ROC | 0.895 | 0.018 | 47.68 | <0.001 | 0.859 | 0.932 |
| Apparent slope | 1.063 | 0.068 | 15.73 | <0.001 | 0.930 | 1.200 |
| Apparent CITL | 0.043 | 0.063 | 0.68 | 0.497 | -0.081 | 0.167 |
| Test ROC | 0.897 | 0.003 | 301.18 | <0.001 | 0.891 | 0.903 |
| Test slope | 1.074 | 0.060 | 17.87 | <0.001 | 0.956 | 1.192 |
| Test CITL | 0.048 | 0.065 | 0.73 | 0.464 | -0.080 | 0.175 |

**Appendix 5.** Locally-weighted scatter plot smoothing (LOWESS) and fractional polynomial assessment of linear association between clinical laboratory predictors and log odds of multiple myeloma: (a) hemoglobin, (b) log serum creatinine, (c) log serum globulin, (d) serum calcium, and (e) log alkaline phosphatase


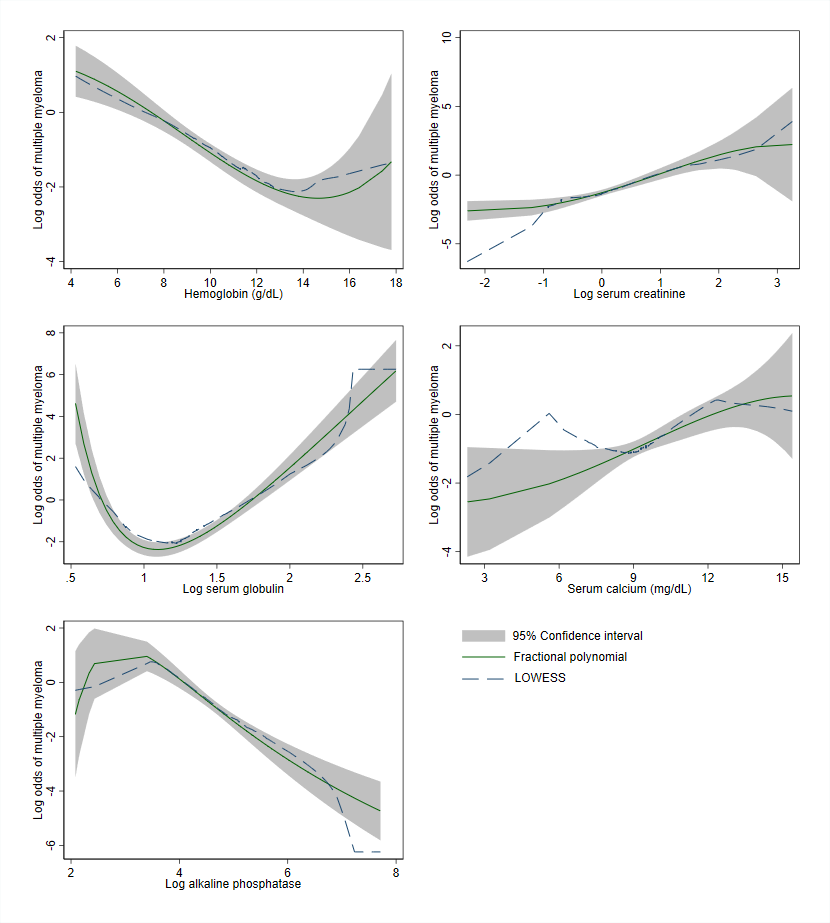

Supplement: Supplementary file 1 — Additional file 1: Appendix 1. International Staging System (ISS) staging of patients with multiple myeloma. Appendix 2. Types of primary cancer in patients diagnosed with bone metastasis. Appendix 3. Closed test algorithm of multivariable fractional polynomial logistic regression model via mfpmi package using an imputed dataset (n = 586). Appendix 4. Internal validation via bootstrap procedures with 100 replicates. Appendix 5. Locally-weighted scatter plot smoothing (LOWESS) and fractional polynomial assessment of linear association between clinical laboratory predictors and log odds of multiple myeloma: (a) hemoglobin, (b) log serum creatinine, (c) log serum globulin, (d) serum calcium, and (e) log alkaline phosphatase. [file 12875_2020_1283_MOESM1_ESM.doc]
